# Phenotypic resistance and virulence gene profiles of non-typhoidal *Salmonella* in retail poultry products in Arusha, Tanzania: A One Health perspective

**DOI:** 10.14202/vetworld.2025.2991-3001

**Published:** 2025-10-08

**Authors:** Salum Ahmed, Ali Ali, Beatus Lyimo, Esther Gwae Kimaro

**Affiliations:** 1 Department of Biomedical Research and Clinical Trial, Ifakara Health Institute (IHI), P.O. Box 74, Bagamoyo, Tanzania; 2 School of Life Science and Bioengineering (LiSBE), The Nelson Mandela African Institution of Science, Technology (NM-AIST), P.O. Box 447, Arusha, Tanzania; 3 Livestock Training Agency (LITA), The Ministry of Livestock and Fisheries, P.O. Box 3101, Arusha, Tanzania

**Keywords:** antimicrobial resistance, non-typhoidal *Salmonella*, One Health, poultry, Tanzania, virulence genes

## Abstract

**Background and Aim:**

Non-typhoidal *Salmonella* (NTS) is a leading cause of foodborne illness, with poultry products serving as major transmission routes. In sub-Saharan Africa, surveillance of antimicrobial resistance (AMR) and virulence determinants remains limited. This study investigated the prevalence, AMR, and virulence gene profiles of NTS isolated from poultry products retailed in Arusha, Tanzania.

**Materials and Methods:**

A cross-sectional study was conducted between August and October 2023. A total of 240 samples (layer eggs and broiler meat) were collected from two wards in Arusha City using systematic random sampling. NTS isolates were confirmed by polymerase chain reaction (PCR) and tested for susceptibility to 11 antimicrobial agents using the Kirby–Bauer method. Virulence (*invA* and *stn*) and resistance genes (*tetA, tetB, bla_TEM_, bla_CTXM_*, and *bla_SHV_*) were screened by PCR. Statistical associations were analyzed using odds ratios (OR) and 95% confidence intervals (CI).

**Results:**

The overall prevalence of NTS was 23.3% (56/240). Layer eggs showed significantly higher contamination (20%) compared with broiler meat (3.3%) (OR = 10.0, 95% CI: 4.4–22.6, p < 0.001). *Salmonella* Typhimurium was the predominant serotype. All isolates carried *invA* and *stn* genes. Alarmingly, 100% of isolates were resistant to imipenem (IMI), while resistance to ampicillin (58.9%) and tetracycline (41.1%) was also common. Multidrug resistance patterns were frequent, although resistance genes were detected at a low prevalence (*tetA*, 5.3%; *bla_TEM_*, 3.5%).

**Conclusion:**

The findings demonstrate a high prevalence of virulent and IMI-resistant *S*. Typhimurium in retail poultry products in Arusha, particularly in eggs. These results highlight critical gaps in food safety regulation and antimicrobial stewardship within the Tanzanian One Health framework. Further genomic studies are warranted to elucidate underlying resistance mechanisms and inform effective surveillance strategies.

## INTRODUCTION

Globally, non-typhoidal *Salmonella* (NTS) is responsible for an estimated 153 million illnesses and over 50,000 deaths annually [1]. It ranks as the second most commonly isolated zoonotic pathogen, accounting for more than 90% of foodborne infections worldwide [2]. NTS is a major cause of gastroenteritis, with poultry products recognized as an important transmission source. Although often self-limiting, salmonellosis exerts a disproportionate impact in sub-Saharan Africa (SSA), which contributes more than 79% of global cases due to its large, vulnerable population [3]. In Tanzania, the estimated incidence was 5.0–19.9 cases/100,000 people in 2017 [4]. Outbreaks of diarrheal disease linked to poultry consumption are frequently associated with *Salmonella* Enteritidis and *Salmonella* Typhimurium [5], and infection typically occurs following ingestion of undercooked or raw poultry products [6]. In Tanzania, salmonellosis is ranked ninth among priority zoonoses under the One Health framework [7], yet reported cases likely represent only a fraction of the true burden [8].

Despite the recognized burden of NTS in SSA, limited studies in Tanzania have combined phenotypic resistance profiling with the molecular detection of virulence and resistance genes in poultry products. Most available reports focus either on prevalence or antimicrobial susceptibility alone, without exploring the coexistence of resistance mechanisms and pathogenic determinants. Furthermore, in northern Tanzania, and particularly in Arusha, a global tourism hub with high poultry product consumption, data on the integrated resistance–virulence profile of NTS are scarce [9]. The absence of such information hampers the design of effective food safety interventions, antimicrobial stewardship strategies, and One Health-based surveillance programs [10, 11].

This study was therefore designed to investigate the prevalence, AMR patterns, and virulence gene profiles of NTS isolated from poultry products retailed in Arusha City, Tanzania. By integrating phenotypic antimicrobial susceptibility testing with molecular detection of key virulence (*invA* and *stn*) and resistance genes (*tetA, tetB, bla_TEM_*, *bla_CTXM_*, and *bla_SHV_*), the study aimed to provide novel insights into the public health risks associated with poultry consumption [12–14]. The findings are expected to inform targeted interventions within the Tanzanian One Health framework and contribute to strengthening local food safety policies.

## MATERIALS AND METHODS

### Ethical approval

This research was approved by the Institutional Review Board of Ifakara Health Institute (IHI/IRB/No: 39-2023). No live animals were involved in the study during sample collection or laboratory analysis. Permission to collect samples under the supervision of public health authorities was granted by the Arusha City local government (Permit No. FA.18/232/002/T/344).

### Study period and location

**Figure 1:**
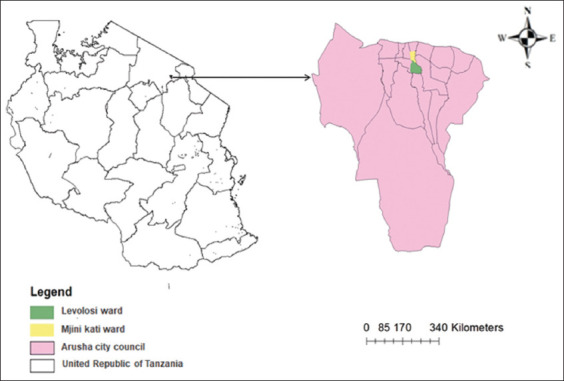
Map of the study area within Tanzania. The left panel highlights the location of Arusha City Council in northern Tanzania (in pink). The right panel provides a detailed view of Arusha City Council, indicating the specific wards involved in the study: Levolosi Ward (green) and Mjini Kati Ward (yellow) [Source: The map was generated using ArcGIS 10.4].

A cross-sectional study was conducted from August to October 2023 in Arusha City, northern Tanzania. Arusha is a key administrative and economic center, recognized as a global tourism hub, situated between latitudes 2°S and 6°S and longitudes 35°E and 38°E. Rapid urbanization and high poultry consumption in this region highlight the public health relevance of the study (Figure 1) [15].

### Sample collection and sampling strategy

Purposive sampling was employed, in consultation with Public health officials to identify retail outlets with the highest turnover of poultry products. Two wards, Mjini Kati (3°22′26′′S, 36°41′18′′E) and Levolosi (3°22′6.6′′S, 36°40′57.72′′E), were selected. Outlets included food stores, groceries, vendors, supermarkets, and open markets.

Systematic sampling was employed by selecting every second store, starting from random points, to minimize bias.

### Sample size estimation

The required sample size was calculated using the formula [16]:


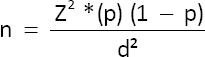


Where:

- n = Sample size
- Z = 1.64 (standard normal deviation at 90% confidence level)
- p = Expected prevalence of zoonotic *Salmonella* at 11.4% [17]
- d = Desired precision (0.05).

The minimum required sample size was 109 per category. To account for spoilage (10%), 120 samples each of broiler meat and eggs were collected, totaling 240 samples.

### Isolation and identification of *Salmonella*

Samples (egg yolk and broiler meat) were pre-enriched in Selenite-F Broth and incubated at 37°C for 24 h. Enriched cultures were streaked onto Xylose-Lysine-Desoxycholate agar, incubated at 37°C for 18–24 h, and examined for colonies with typical morphology (pink colonies with black centers). Colonies were further subjected to Gram staining and microscopic observation at 1,000× magnification to confirm rod-shaped Gram-negative bacteria consistent with *Salmonella* [18].

### Molecular confirmation by polymerase chain reaction (PCR)

DNA was extracted using the boiling lysis method. Each isolate was suspended in 200 μL of broth, centrifuged at 14,000 × *g*, resuspended in sterile water, and then subjected to heating at 100°C for 10 min, followed by rapid cooling and a second centrifugation step. The resulting supernatant served as PCR template.

PCR reactions (25 μL) contained: 12.5 μL OneTaq Quick-Load 2× Master Mix (New England Biolabs, Inc. MA, USA, Catalog No.: M0486S/M0486L), 1 μL forward and reverse primers, 4 μL DNA template, and 6.5 μL nuclease-free water (New England Biolabs, USA) (Table 1) [19, 20].

**Table 1:** Primers used to confirm NTS infection.

S. No.	Target gene	Strain name	Primer set	Primer sequence (5’––3’)	Primer length	Band size (bp)	Reference
1.	*sefA*	*Salmonella* Enteritidis	S1/S4	F: GCCGTACACGAGCTTATAGA R: ACCTACAGGGGCACAATAAC	20	250	[19]
2.	*fliC*	*Salmonella* Typhimurium	Fli15/Typ04	F: CGGTGTTGCCCAGGTTGGTAAT R: ACTCTTGCTGGCGGTGCGACTT	22	559	[20]

F = Forward, R = Reverse, bp = Base pair, NTS = Non-typhoidal *Salmonella*, *sefA* = S*almonella* Enteritidis fimbrial A gene, *fliC* = Flagellin C gene, encodes phase 1 H antigen

Amplification was performed using a Bio-Rad C1000 Touch Thermal Cycler (Bio-Rad, CA, USA):

- Initial denaturation: 94°C for 30 s
- 34 cycles of: 94°C for 30 s (denaturation), 50°C for 30 s (*S*. Enteritidis) or 57°C (*S*. Typhimurium) for annealing, and 68°C for 1 min (extension)
- Final extension: 68°C for 5 min.

Products were resolved on 2% agarose gel electrophoresis, stained with ethidium bromide, and visualized under a ChemiDoc MP system (Bio-Rad). Positive controls (*S*. Enteritidis American Type Culture Collection [ATCC] 13076; *S*. Typhimurium ATCC 14028) and a negative control were included in the study.

### Antimicrobial susceptibility testing

Antimicrobial susceptibility was tested using the Kirby–Bauer disc diffusion method, following Clinical and Laboratory Standards Institute (CLSI) (2018) guidelines [21]. Standardized inocula (1.5 × 10^8 colony-forming unit/mL; McFarland standard) were spread onto Mueller–Hinton agar (HiMedia, India). Antibiotic discs (Acon Laboratories Ltd. San Diego, California, USA) included: ampicillin (AMP) (25 μg), cefaclor (CF) (30 μg), imipenem (IMI) (10 μg), gentamycin (GEN) (10 μg), ciprofloxacin (CIP) (5 μg), sulfamethoxazole-trimethoprim (SXT) (25 μg), streptomycin (S) 10 μg, chloramphenicol (C) (30 μg), doxycycline (DXT) (30 μg), norfloxacin (NOR) (10 μg), and TE (30 μg). Plates were incubated at 37°C for 18–25 h, and inhibition zones were interpreted using CLSI breakpoints. *S*. Typhimurium ATCC 14028 served as quality control.

### Detection of virulence and resistance genes

DNA from confirmed *S*. Typhimurium isolates was subjected to PCR for virulence (*invA* and *stn*) and resistance genes (*tetA*, *tetB*, *bla_TEM_*, *bla_CTXM_*, and *bla_SHV_*) (Tables 2 and 3) [22, 23].

**Table 2:** Primer pairs for detecting virulence genes.

S. No.	Target gene	Primers (5’––3’)	Annealing temperature	Band size (bp)	Reference
1.	*stn*	F: TTGTGTCGCTATCACTGG CAACC R: ATT CGT AAC CCG CTC TCG TCC	60.5°C	617	[22]
2.	*invA*	F: TAT CGC CAC GTT CGG CAA R: TCG CAC CGT CAA AGG AAC C	60.5°C	275	[22]

F = Forward, R = Reverse, bp = Base pair, *stn* = S*almonella* enterotoxin gene, *invA* = Invasion protein A gene

**Table 3:** Primer pairs for detecting AMR genes.

Drug type	Gene name	Primer (5’––3’)	Annealing temp	Base pairs	Reference
Tetracycline	*tetA*	F: GCTACATCCTGCTTGCCTTC R: CATAGATCGCCGTGAAGAGG	54°C	210	[22]
	*tetB*	F: TTGGTTAGGGGCAAGTTTTG R: GTAATGGGCCAATAACACCG	52°C	659	[22]
ESBL	*bla_TEM_*	F: TTGGGTGCACGAGTGGGTTA R: TAATTGTTGCCGGGAAGCTA	57°C	465	[23]
	*bla_SHV_*	F: AGGATTGACTGCCTTTTTG R: ATTTGCTGATTTCGCTCG3	60°C	392	[23]
	*bla_CTXM_*	F: ACCGCCGATAATTCGCAGAT R: GATATCGTTGGTGGTGCCATAA	57°C	588	[23]

AMR = Antimicrobial resistance, ESBL = Extended-spectrum beta-lactamase, F = Forward, R = Reverse, *tetA* = Tetracycline resistance genes type A, *tetB* = Tetracycline resistance gene type B, *bla_TEM_* = β-lactamase TEM-type gene, *bla_SHV_* = β-lactamase SHV-type gene, *bla_CTXM_* = β-lactamase *_CTX-M_*-type gene

- Virulence gene PCR: Initial denaturation at 95°C for 1 min; 34 cycles of 95°C for 20 s, 60.5°C for 1 min (annealing), and 68°C for 1 min (extension); final extension at 68°C for 5 min.
- Resistance gene PCR: 40 cycles of 94°C for 30 s, annealing at gene-specific temperatures (54°C *tetA*, 52°C *tetB*, 57°C *bla_TEM/CTXM_*, and 60°C *bla_SHV_*), extension at 68°C for 1 min, and final extension at 68°C for 5 min.

Amplified products were visualized on agarose gel under the same electrophoresis conditions as Section 2.5 [24].

### Statistical analysis

Data were entered in Microsoft Excel and analyzed using Stata v15 (StataCorp, USA). Descriptive statistics (frequencies, percentages, tables, and graphs) summarized prevalence and resistance profiles. Associations between sample type, ward, and contamination were assessed using odds ratios (ORs) with 95% confidence intervals (CIs). The Chi-square test was used to determine significance, with p-value set at < 0.05.

## RESULTS

### Frequency of NTS detection

The overall prevalence of NTS in poultry products was 23.3% (56/240). Layer eggs showed the highest contamination (20%) compared with broiler meat (3.3%). No *S*. Enteritidis was detected in any of the samples (Table 4). *S*. Typhimurium was the predominant serovar, detected across all sampling points, with prevalence ranging from 0.4% (1/10) to 4.2% (10/50).

**Table 4:** Frequency of NTS detected from raw chicken products in the Arusha City Council.

Wards	Sampling points	Sample size	No. of positive samples (%)		Total number of isolates

			*Salmonella* Typhimurium	*Salmonella* Enteritidis	
Levolosi	Katimakutano	14	2 (0.8)	0	20 (16.7)
	Kilombelo	33	5 (2.0)	0	
	Marombosa	24	1 (0.4)	0	
	Salei	10	1 (0.4)	0	
	Shoppers	28	9 (3.8)	0	
	Soka	11	2 (0.8)	0	
Mjini kati	Majengo	22	9 (3.8)	0	36 (30)
	Ndovu	19	10 (4.2)	0	
	Swahili	16	2 (0.8)	0	
	Sokokuu	50	7 (2.9)	0	
	Wachaga	13	8 (3.3)	0	
	Total	240	56 (23.3)	0	56 (23.3)

NTS = Non-typhoidal *Salmonella*

**Figure 2:**
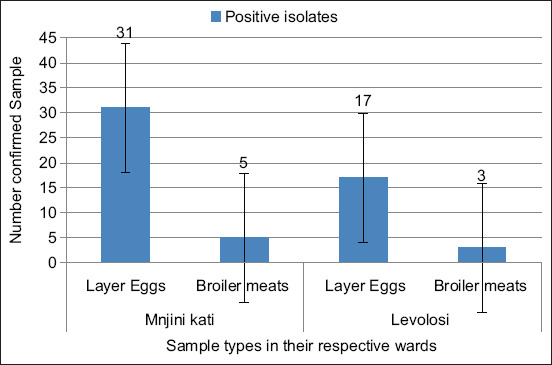
Contamination of the *Salmonella* Typhimurium was detected in the egg sample (48 isolates) and in the meat samples (8 isolates).

The highest contamination levels were observed at Sokokuu (10/50; 4.2%), Majengo (9/22; 3.8%), and Wachaga (8/13; 3.3%). Eggs accounted for most isolates (48/56), while broiler meat contributed only 8 isolates (Figure 2). Error bars (±12.9 standard deviation) indicated considerable variability in prevalence estimates.

Geographically, Mjini Kati ward recorded a prevalence of 15% (36/240), which was significantly higher than Levolosi ward at 8.3% (20/240). Statistical analysis showed that eggs were 10 times more likely to be contaminated with *Salmonella* than meat (OR = 10.0; 95% CI: 4.399–22.602; p < 0.001). Similarly, samples from Mjini Kati were 2.5 times more likely to be contaminated compared with those from Levolosi (OR = 2.474; 95% CI: 1.257–4.867; p = 0.009) (Table 5).

**Table 5:** Predictor of NTS contamination.

Predictor variable	Frequency (n)	OR	95% CI	p-value
Sample type	240	10.0	4.4–22.6	<0.001
Ward	240	2.5	1.3–4.9	0.009

NTS = Non-typhoidal *Salmonella*, OR = Odds ratio, CI = Confidence interval

### Antibiotic susceptibility profile of *S*. Typhimurium isolates

**Figure 3:**
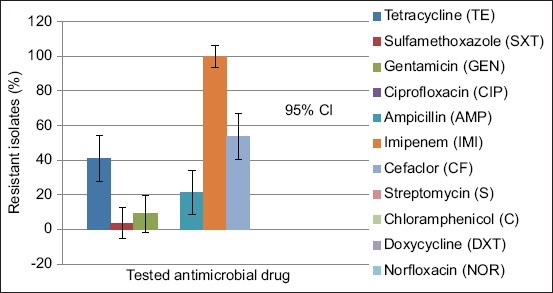
Antibiotic drugs used in antimicrobial sensitivity against *Salmonella* Typhimurium were imipenem, cefaclor, tetracycline, sulfamethoxazole-trimethoprim, ampicillin, gentamycin, streptomycin, ciprofloxacin, doxycycline, norfloxacin, and chloramphenicol.

Resistance levels varied across antimicrobial agents (Figure 3). All isolates (100%) were resistant to IMI. Moderate resistance was observed against AMP (58.9%) and TE (41.1%), while resistance to CF was detected in 21.4% of isolates. Lower resistance rates were recorded for GEN and SXT, whereas no resistance was observed against CIP, NOR, S, C, or DXT.

Resistance patterns are summarized in Table 6. Of the 56 isolates, 23 (41.1%) were monoresistant to IMI. Combined resistance to IMI–CF was observed in 9 isolates (16.1%), while the TE–AMP–IMI–CF pattern was found in 8 isolates (14.3%). Three isolates (5.4%) showed multidrug resistance to TE, GEN, AMP, IMI, and CF. Less common resistance profiles included TE–SXT–IMI–CF and TE–SXT–GEN–IMI–CF (one isolate each).

**Table 6:** Drug combinations resistant to *Salmonella* Typhimurium.

S. No.	Resistance profile	No. of resistant isolates (%)	Status
1.	AMP-IMI-CF	1 (1.8)	Multi-resistant
2.	GEN-IMI-CF	1 (1.8)	Multi-resistant
3.	IMI	23 (41.1)	Mono-resistant
4.	IMI-CF	9 (16.1)	Multi-resistant
5.	TE-AMP-IMI-CF	8 (14.3)	Multi-resistant
6.	TE-GEN-AMP-IMI-CF	3 (5.4)	Multi-resistant
7.	TE-IMI	3 (5.4)	Multi-resistant
8.	TE-IMI-CF	6 (10.7)	Multi-resistant
9.	TE-SXT-GEN-IMI-CF	1 (1.8)	Multi-resistant
10.	TE-SXT-IMI-CF	1 (1.8)	Multi-resistant
11.	Total	56	

IMI = Imipenem, CF = Cefaclor, TE = Tetracycline, SXT = Sulfamethoxazole-trimethoprim, AMP = Ampicillin, GEN = Gentamycin

### Antibiotic resistance and virulence genes of *Salmonella* isolates

**Figure 4:**
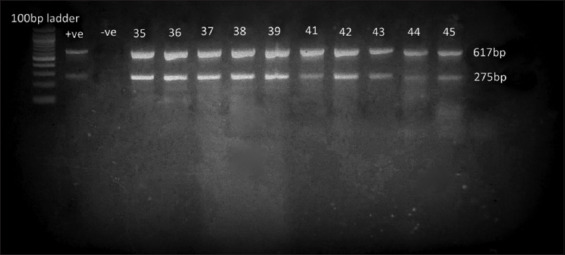
Electrophoresis gel image showing bands corresponding to the *invA* and *stn* virulence genes, amplified using multiplex polymerase chain reaction, with expected band sizes of 275 bp and 617 bp, respectively. Well 1 contains the positive control, and well 2 contains the negative control. The remaining wells showed positive amplification for both *invA* and *stn*.

**Figure 5:**
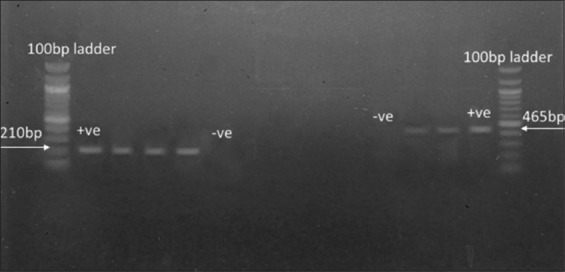
Raw electrophoresis gel image showing *tetA* and *bla_TEM_* resistance genes using single-plex amplification. The *tetA* gene was detected in wells 2, 3, and 4, with positive and negative controls in wells 2 and 6, respectively. The *bla_TEM_* gene was observed in samples from wells 13 and 14, and the positive and negative controls were located in wells 11 and 12, respectively.

Molecular analysis revealed that all 56 isolates carried the virulence genes *invA* and *stn* (Figure 4), with 100% prevalence in both egg and meat samples. In contrast, most resistance genes screened (*tetA*, *tetB*, *bla_TEM_*, *bla_SHV_*, and *bla_CTXM_*) were absent. Only three isolates carried *tetA* and three carried *bla_TEM_*. The *tetA* gene was detected mainly in egg isolates, while *bla_TEM_* was found in both eggs and broiler meat (Figure 5).

## DISCUSSION

### Public Health concern of NTS in SSA

The prevalence of NTS infections associated with poultry products is a major public health concern in SSA [25]. This study confirmed contamination in both raw layer eggs and broiler meat in Arusha, Tanzania, highlighting the zoonotic potential of these bacteria. The findings align with earlier reports of high prevalence in developing countries, particularly in urban centers such as Arusha, where weak infrastructure and inadequate social services hinder the implementation of food safety measures [26]. Rapid population growth and urbanization contribute to inequalities in food handling systems [27], thereby increasing risks of contamination in the poultry supply chain [28].

### Contamination patterns in eggs and meat

The high contamination rate (23.3%) of *S*. Typhimurium underscores the role of external contamination pathways, given its ability to survive in diverse hosts and environments. Unlike *S*. Enteritidis, which can contaminate eggs internally [29], *S*. Typhimurium contamination is linked primarily to handling and processing practices. These findings are consistent with regional studies where *S*. Enteritidis is typically associated with animal products, whereas *S*. Typhimurium is more frequently linked to human infections but both serovars cross those lines a lot, and the picture varies by food chain practices and surveillances within SSA [30, 31].

The significantly higher contamination in raw eggs (20%) compared with broiler meat (3.3%) suggests that eggs represent a more critical reservoir for zoonotic *Salmonella* [32]. Statistical analysis confirmed a strong association between egg contamination and salmonellosis risk (p < 0.0001; 95% CI: 4.298–21.823). Importantly, eggs are often consumed raw or undercooked in products such as mayonnaise, which increases the risk of foodborne outbreaks [33], unlike meat, which generally undergoes thorough cooking [34]. Informal markets, where eggs are sold without standardized handling procedures, likely amplify this risk.

### Geographical disparities in contamination

Contamination levels varied between Mjini Kati ward (15%) and Levolosi ward (8.3%). Although the association with geographical location was weaker (p = 0.048), the variation may reflect differences in local market practices, hygiene standards, and regulatory oversight. Poor temperature control and unhygienic handling during poultry processing likely contribute to elevated contamination rates in certain locations [35]. This emphasizes the need for targeted public health interventions, particularly in high-risk urban wards [36].

### Antibiotic resistance trends

The antibiotic susceptibility profile revealed distinct resistance patterns. While many isolates remained susceptible to several commonly used antibiotics, the detection of multidrug-resistant *S*. Typhimurium is alarming. All isolates (100%) exhibited resistance to IMI, with 41.1% showing monoresistance and 58.9% demonstrating multidrug resistance. This suggests that IMI has effectively lost its therapeutic value against *Salmonella* in the study area. These findings are consistent with earlier studies documenting IMI-resistant bacterial populations in northern Tanzania’s poultry sector [37].

The widespread resistance to IMI represents a serious public health threat, raising concerns about interspecies transmission and dissemination across the food chain. Cross-border livestock movement, coupled with the absence of traceability systems, increases the risk of spreading carbapenem-resistant strains to broader regions [38]. Continuous surveillance and stricter antimicrobial stewardship are urgently required to address these challenges [39].

### Multidrug resistance profiles

The isolates exhibited varied resistance combinations, including resistance to CF, TE, GEN, SXT, and AMP. Multi-resistance patterns such as TE–AMP–IMI–CF (14.3%) and TE–GEN–AMP–IMI–CF (5.4%) complicate treatment options for salmonellosis. Horizontal gene transfer may play a key role in the dissemination of these resistance traits. Despite this, susceptibility to drugs such as S, CIP, DXT, NOR, and C provides alternative therapeutic options that must be preserved through prudent use [40].

### Virulence and resistance genes

The detection of virulence genes (*invA* and *stn*) in 100% of isolates indicates high pathogenic potential. These genes enhance the ability of *Salmonella* to *inva*de host intestinal cells and produce enterotoxins, thereby increasing the risk of severe clinical salmonellosis [41], especially in vulnerable populations such as children, pregnant women, the elderly, and immunocompromised individuals. The universal presence of these genes underscores the likelihood of diarrheal outbreaks if food safety measures are not improved [42].

Resistance gene screening revealed a low prevalence of *tetA* (5.3%) and *bla_TEM_* (3.5%). *tetA* was detected primarily in egg isolates and correlated with phenotypic tetracycline (TE) resistance, while *bla_TEM_* was associated with AMP and cefaclor resistance. Interestingly, phenotypic resistance was observed even in isolates lacking these genes, suggesting alternative mechanisms such as efflux pumps or other adaptive strategies. The low prevalence of resistance genes contrasts with findings from other East African studies [43], which reported higher rates of *tetA*, indicating possible geographic variation in gene dissemination [44].

### Limitations

This study provides valuable insights into the prevalence, resistance, and virulence profiles of NTS in Arusha poultry products; however, certain limitations must be acknowledged. The absence of *S*. Enteritidis may be attributed to molecular limitations, such as primer mismatches with local strains. The cross-sectional design, limited to two wards, restricts the ability to capture temporal and broader spatial variations. Furthermore, while high phenotypic resistance to IMI was observed, the absence of molecular confirmation for carbapenemase genes limits interpretation of underlying mechanisms. Future investigations employing whole-genome sequencing and minimum inhibitory concentration (MIC) assays are recommended for more robust analysis.

## CONCLUSION

This study revealed a high prevalence (23.3%) of NTS in poultry products retailed in Arusha City, with *S*. Typhimurium as the predominant serovar. Contamination was markedly higher in layer eggs (20%) compared with broiler meat (3.3%), indicating that eggs serve as a critical reservoir for zoonotic transmission. All isolates carried the virulence genes *invA* and *stn*, confirming their high pathogenic potential. Alarmingly, 100% of isolates demonstrated resistance to IMI, while multidrug resistance patterns involving AMP, TE, and CF further underscored the treatment challenges. Although resistance genes such as *tetA* and *bla_TEM_* were detected at a low prevalence, the presence of phenotypic resistance in their absence suggests that alternative mechanisms are at play.

These findings emphasize the urgent need for strengthened food safety policies, particularly in informal poultry markets where eggs are handled with minimal hygienic control. The demonstrated IMI resistance highlights a potential loss of a critical therapeutic option, necessitating prudent antimicrobial stewardship across both veterinary and human health sectors. Public education on safe handling and thorough cooking of poultry products is vital to reduce transmission risks.

This study is among the first in northern Tanzania to integrate phenotypic resistance profiling with molecular detection of virulence and resistance genes in poultry products, providing a comprehensive risk assessment under the One Health framework.

Future research should employ whole-genome sequencing and MIC assays to elucidate resistance mechanisms and track gene dissemination. Expanded surveillance across Tanzania and integration of poultry supply chain traceability systems are needed to enhance early detection and response to resistant *Salmonella*.

The coexistence of high virulence potential and alarming AMR in *S*. Typhimurium from poultry products represents a significant public health threat in Arusha. Targeted surveillance, improved hygiene practices, and stronger antimicrobial stewardship anchored in the One Health framework are essential to safeguard food safety and preserve the efficacy of critical antibiotics.

## AUTHORS’ CONTRIBUTIONS

EGK and SA: Conceptualization and design of the study. SA: Sample and data collection. AA: Data analysis and interpretation. BL: Laboratory work and data acquisitions. All authors have read and approved the final manuscript.

## References

[1] Plumb I, Fields, Patricia and Bruce B (CDC) (2024). Salmonellosis, Nontyphoidal. In CDC Yellow Book 2024:Health Information for International Travel. Centers for Disease Control and Prevention.

[2] FAO and WHO (2023). Measures for the Control of Non-typhoidal *Salmonella* spp. Poultry Meat. Meas Control non-Typhoidal *Salmonella* spp Poult Meat. FAO, Rome.

[3] James S.L, Abate D, Abate K.H, Abay T, Murray C.J.L (2018). Global, regional, and national incidence, prevalence, and years lived with disability for 354 diseases and injuries for 195 countries and territories, 1990-2017:A systematic analysis for the Global Burden of Disease Study 2017. Lancet.

[4] Stanaway J.D, Parisi A, Sarkar K, Blacker B.F, Reiner B.X, Ullah I, Yimer E.M, Zaidi Z, Murray C.J.L, Crump J.A (2019). The global burden of non-typhoidal *Salmonella* invasive disease:A systematic analysis for the Global Burden of Disease Study 2017. Lancet Infect. Dis.

[5] EFSA and ECDC (2020). Multi-country outbreak of *Salmonella* Enteritidis infections linked to eggs, third update –6 February 2020. EFSA Support Publ.

[6] Heredia N, García S (2018). Animals as sources of food-borne pathogens:A review. Anim. Nutr.

[7] Department of Health and Human Services (2017). CDC, USAID and FAO. One Health Zoonotic Disease Prioritization for Multisectoral Engagement in Tanzania.

[8] Ngogo F.A, Joachim A, Abade A.M, Rumisha S.F, Mizinduko M.M, Majigo M.V (2020). Factors associated with *Salmonella* infection in patients with gastrointestinal complaints seeking health care at Regional Hospital in Southern Highland of Tanzania. BMC Infect. Dis.

[9] Sonola V.S, Katakweba A, Misinzo G, Matee M.I (2022). Molecular epidemiology of antibiotic resistance genes and virulence factors in multidrug-resistant *Escherichia coli* isolated from rodents, humans, chicken, and household soils in Karatu, Northern Tanzania. Int. J. Environ. Res. Public Health.

[10] Escher N.A, Muhummed A.M, Hattendorf J, Vonaesch P, Zinsstag J (2021). Systematic review and meta-analysis of integrated studies on antimicrobial resistance genes in Africa-A One Health perspective. Trop. Med. Int. Health.

[11] Bertagnolio S, Dobreva Z, Centner C.M, Olaru I.D, Donà D, Burzo S, Huttner B.D, Chaillon A, Gebreselassie N, Wi T, Hasso-Agopsowicz M, Allegranzi B, Sati H, Ivanovska V, Kothari K.U, Balkhy H.H, Cassini A, Hamers R.L, Weezenbeek K.V, WHO Research Agenda for AMR in Human Health Collaborators (2024). WHO global research priorities for antimicrobial resistance in human health. Lancet Microbe.

[12] World Health Organization (2022). Global Antimicrobial Resistance and Use Surveillance System (GLASS).

[13] Islam M.S, Sobur M.A, Rahman S, Ballah F.M, Ievy S, Siddique M.P, Rahman M, Kafi M.A, Rahman M.T (2022). Detection of *bla_TEM_*, *bla***_CTX-M_**, *bla_CMY_*, and *bla_SHV_* genes among extended-spectrum beta-lactamase-producing *Escherichia coli* isolated from migratory birds travelling to Bangladesh. Microb. Ecol.

[14] Kiula A.H, Makene V.A (2023). Molecular epidemiology of antibiotic resistance among *Escherichia coli* isolated from broiler chickens sold at selected markets in dar es Salaam, Tanzania. Tanzania J. Sci.

[15] Kessy D, Kiage O, Kiprutto N (2018). Multiplier effects of tourism in selected areas of Arusha, Tanzania. African J. Hosp. Tour. Leis.

[16] Lwanga S.K, Lemeshow S (1991). Sample Size Determination in Health Studies.

[17] Sindiyo E, Missanga J (2018). Common diseases affecting poultry production in Arusha Peri-urban, Northern Tanzania. J. Appl. Life Sci. Int.

[18] Siddique A, Azim S, Ali A, Andleeb S, Ahsan A, Imran M, Rahman A (2021). Antimicrobial resistance profiling of biofilm forming non typhoidal *Salmonella enterica* isolates from poultry and its associated food products from Pakistan. Antibiotics (*Basel*).

[19] Krzyzanowski F, Zappelini L, Martone-Rocha S, Dropa M, Matté M.H, Nacache F, Razzolini M.T.P (2014). Quantification and characterization of *Salmonella* spp. Isolates in sewage sludge with potential usage in agriculture. BMC Microbiol.

[20] Mendybayeva A, Abilova Z, Bulashev A, Rychshanova R (2023). Prevalence and resistance to antibacterial agents in *Salmonella enterica* strains isolated from poultry products in Northern Kazakhstan. Vet. World.

[21] CLSI (2018). Performance Standards for Antimicrobial Susceptibility Testing.

[22] Diab M.S, Thabet A.S, Elsalam M.A, Ewida R.M, Sotohy S.A (2023). lactamase resistance genes of non-typhoidal *Salmonella* isolates from human and animal origin in Egypt “one health concern”. Gut Pathog.

[23] Wang M.Y, Geng J.L, Chen Y.J, Song Y, Sun M, Liu H.Z, Hu C.J (2017). Direct detection of mecA, *bla_SHV_,*
*bla_CTX-M_*, *bla_TEM_* and *bla_OXA_* genes from positive blood culture bottles by multiplex-touchdown PCR assay. Lett. Appl. Microbiol.

[24] Kong-Ngoen T, Santajit S, Tunyong W, Pumirat P, Sookrung N, Chaicumpa W, Indrawattana N (2022). Antimicrobial resistance and virulence of non-typhoidal *Salmonella* from retail foods marketed in Bangkok, Thailand. Foods.

[25] Tack B, Vanaenrode J, Verbakel J.Y, Toelen J, Jacobs J (2020). Invasive non-typhoidal *Salmonella* infections in sub-Saharan Africa:A systematic review on antimicrobial resistance and treatment. BMC Med.

[26] Fisher R, Currie P (2019). Towards a safe, nourishing, economic and inclusive food system for Arusha, based on Partnering.

[27] Mramba R.P, Mwantambo P.A (2024). The impact of management practices on the disease and mortality rates of broilers and layers kept by small-scale farmers in Dodoma urban district, Tanzania. Heliyon.

[28] Archer E.W, Chisnall T, Tano-Debrah K, Card R.M, Duodu S, Kunadu A.P.H (2023). Prevalence and genomic characterization of *Salmonella* isolates from commercial chicken eggs retailed in traditional markets in Ghana. Front. Microbiol.

[29] Crump J.A, Thomas K.M, Benschop J, Knox M.A, Wilkinson D.A, Midwinter A.C, Munyua P.C, Ochieng J.B, Bigogo G.M, Verani J.R, Widdowson M.A, Prinsen G, Cleaveland S, Karimuribo E.D, Kazwala R.R, Mmbaga B.T, Swai E.S, French N.P, Zadoks R.N (2021). Investigating the meat pathway as a source of human nontyphoidal *Salmonella* bloodstream infections and diarrhea in East Africa. Clin. Infect. Dis.

[30] Kamboj S, Gupta N, Bandral J.D, Gandotra G, Anjum N (2020). Food safety and hygiene:A review. Int. J. Chem. Stud.

[31] Kivali V, Roesel K, Dohoo I, Alinaitwe L, Bugeza J.K, Hoona J.J, Mugizi D.R, Kankya C, Dang-Xuan S, Szabo I, Rösler U, Friese A, Cook E.A.J (2024). Non-typhoidal *Salmonella* among slaughterhouse workers and in the pork value chain in selected districts of Uganda. Front. Vet. Sci.

[32] Moffatt C.R.M, Musto J, Pingault N, Miller M, Stafford R, Gregory J, Polkinghorne B.G, Kirk M.D (2016). *Salmonella* Typhimurium and outbreaks of egg-associated disease in Australia, 2001 2011. Foodborne Pathog. Dis.

[33] Hernández-Olivas E, Muñoz-Pina S, Andrés A, Heredia A (2021). Impact of cooking preparation on *in vitro* digestion of eggs simulating some gastrointestinal alterations in elders. J. Agric. Food Chem.

[34] Lianou A, Panagou E.Z, Nychas G.J.E (2017). Meat safety-I foodborne pathogens and other biological issues. In:Lawrie's Meat Science.

[35] Garridogamarro E, Svanevik C.S, Lundebye A.K, Sanden M, D'Agostino E, Kjellevold M, Pincus L, Pucher J (2023). Challenges in the implementation of food safety and quality assurance systems in small-scale fisheries. Food Qual. Saf.

[36] Aga A.M, Mulugeta D, Muleta D, Woldesemayat A.A, Motuma A, Kelel M, Wakitole B, Tadesse S, Teferi Z, Woldemariyam F.T, Oljira S, Ferede H, Berihun N, Girma S, Nigussie D, Gemeda M.T (2025). Antibiotic susceptibility patterns of *Salmonella* isolates from clinical, food, and environmental sources in Addis Ababa and surrounding towns, Ethiopia. Microbiol. Spectr.

[37] Masoud S.S, Njakoi G.N, Sholla S, Renatus D, Majigo M, Gangji R.R, Nyawale H, Mawazo A, Ntukula A, Kamori D (2024). Carbapenem resistance in *Pseudomonas aeruginosa* and *Acinetobacter baumannii* in Tanzania. Ger. J. Microbiol.

[38] Mutua F, Kihara A, Rogena J, Ngwili N, Aboge G, Wabacha J, Bett B (2018). Piloting a livestock identification and traceability system in the northern Tanzania-Narok-Nairobi trade route. Trop. Anim. Health Prod.

[39] Ssekatawa K, Byarugaba D.K, Wampande E, Ejobi F (2018). A systematic review:The current status of carbapenem resistance in East Africa. BMC Res. Notes.

[40] Katale B.Z, Misinzo G, Mshana S.E, Chiyangi H, Campino S, Clark T.G, Good L, Rweyemamu M.M, Matee M.I (2020). Genetic diversity and risk factors for the transmission of antimicrobial resistance across human, animals and environmental compartments in East Africa:A review. Antimicrob. Resist. Infect. *Control*.

[41] Shalal S.H, Jaber N.N, Hussein K.R (2023). Molecular identification of virulence genes *Salmonella enterica* isolated of animal and human diarrheal. J. Med. Chem. Sci.

[42] Khalefa H.S, Ahmed Z.S, Abdel-Kader F, Ismail E.M, Elshafiee E.A (2021). Sequencing and phylogenetic analysis of the stn gene of *Salmonella* species isolated from different environmental sources at Lake Qarun protectorate:The role of migratory birds and public health importance. Vet. World.

[43] Onduru O.G, Mkakosya R.S, Aboud S, Rumisha S.F (2021). Genetic determinants of resistance among ESBL-producing Enterobacteriaceae in community and hospital settings in East, Central, and Southern Africa:A systematic review and meta-analysis of prevalence. Can. J. Infect. Dis. Med. Microbiol.

[44] Lund D, Parras-Moltó M, Inda-Díaz J.S, Ebmeyer S, Larsson D.G.J, Johnning A, Kristiansson E (2025). Genetic compatibility and ecological connectivity drive the dissemination of antibiotic resistance genes. Nat. Commun.

